# Resting-state EEG features modulated by depressive state in healthy individuals: insights from theta PSD, theta-beta ratio, frontal-parietal PLV, and sLORETA

**DOI:** 10.3389/fnhum.2024.1384330

**Published:** 2024-08-12

**Authors:** Pengcheng Li, Mio Yokoyama, Daiki Okamoto, Hironori Nakatani, Tohru Yagi

**Affiliations:** ^1^School of Environment and Society, Tokyo Institute of Technology, Tokyo, Japan; ^2^School of Information and Telecommunication Engineering, Tokai University, Tokyo, Japan; ^3^School of Engineering, Tokyo Institute of Technology, Tokyo, Japan

**Keywords:** subthreshold depression, electroencephalogram (EEG), resting state, power spectrum density (PSD), phase-locking value (PLV), source localization, sLORETA

## Abstract

Depressive states in both healthy individuals and those with major depressive disorder exhibit differences primarily in symptom severity rather than symptom type, suggesting that there is a spectrum of depressive symptoms. The increasing prevalence of mild depression carries lifelong implications, emphasizing its clinical and social significance, which parallels that of moderate depression. Early intervention and psychotherapy have shown effective outcomes in subthreshold depression. Electroencephalography serves as a non-invasive, powerful tool in depression research, with many studies employing it to discover biomarkers and explore underlying mechanisms for the identification and diagnosis of depression. However, the efficacy of these biomarkers in distinguishing various depressive states in healthy individuals and in understanding the associated mechanisms remains uncertain. In our study, we examined the power spectrum density and the region-based phase-locking value in healthy individuals with various depressive states during their resting state. We found significant differences in neural activity, even among healthy individuals. Participants were categorized into high, middle, and low depressive state groups based on their response to a questionnaire, and eyes-open resting-state electroencephalography was conducted. We observed significant differences among the different depressive state groups in theta- and beta-band power, as well as correlations in the theta–beta ratio in the frontal lobe and phase-locking connections in the frontal, parietal, and temporal lobes. Standardized low-resolution electromagnetic tomography analysis for source localization comparing the differences in resting-state networks among the three depressive state groups showed significant differences in the frontal and temporal lobes. We anticipate that our study will contribute to the development of effective biomarkers for the early detection and prevention of depression.

## 1 Introduction

Major depressive disorder (MDD) is a pervasive mental health challenge that affects over 322 million people globally, and its prevalence tends to increase with age.^1^ Characterized by profound alterations in mood, cognitive function, and overall well-being, MDD not only diminishes individual quality of life but also imposes a significant societal burden. The complexity of MDD and its enormous impact highlight the urgent need for reliable methods of early diagnosis. Depressive states in healthy individuals and those with MDD differ quantitatively, but not qualitatively (Cox et al., 2001). The qualitative differences refer to different types of expression, such as psychological experiences and depressive symptoms. Meanwhile, quantitative differences refer to differences in the degree of depression or severity of symptoms rather than the type of symptoms (Flett et al., 1997). Previous studies have found a continuum of depressive symptoms through cluster analysis, where the depressive state is on a continuum or spectrum, and as the severity of a symptom increases, so too does the likelihood that it will be categorized as depressive in the cluster analysis. Individuals with mild depression are more likely to develop moderate depression than those without depression, and psychotherapy has been proven to be effective in treating subclinical depression (Cuijpers et al., 2014). The prevalence of mild depression is on a gradual upward trend and has lifelong implications (An et al., 2022). Mild depression is no less socially and clinically significant than moderate depression, and early intervention in subthreshold depression is more likely to yield a positive impact on adolescents (Cuijpers et al., 2021).

Electroencephalography (EEG) holds promise in the diagnosis and management of depression, with potential applications in treatment selection, classification, and prediction of the treatment response. Moreover, EEG has been suggested to assist in differentiating patients with depression from those with other clinical disorders and from normal individuals (Jaseja, 2023). A previous study demonstrated the effectiveness of power spectrum density (PSD) in analyzing EEG signals for early-stage depression, offering a deeper understanding of brain activities under different physiological conditions (Grin-Yatsenko et al., 2009). Several studies have reported differences in EEG power between patients with MDD and healthy individuals. Increased delta-band phase lag and reduced resting-state gamma current density in the anterior cingulate cortex have been found in MDD patients (Simmatis et al., 2023). Another study found that MDD patients showed significantly elevated current density in delta, theta, alpha, beta1, and beta2 frequency bands relative to controls in the anterior cingulate and prefrontal cortices (Korb et al., 2008). Additionally, it has been reported that the frontal gamma power in individuals with depression is increased relative to healthy controls, and that delta power during sleep is lower in individuals with depression than in healthy controls (Meerwijk et al., 2015). Analysis of EEG power ratios has also shown significant differences in specific ratios between depressed patients and healthy controls, including the alpha–beta ratio (ABR) and the theta–beta ratio (TBR), suggesting that these ratios could be used as biomarkers of depression (Chang and Choi, 2023).

In clinical neuroscience, the phase locking value (PLV) has been a valuable tool for identifying aberrant neural connectivity patterns in various neurological disorders, offering new perspectives on the pathophysiology of these disorders (Uhlhaas and Singer, 2006). Previous studies have explored the relationship between EEG patterns and changes in depressive mood, and found that individuals’ changes in depressive mood over certain consecutive time periods were associated with resting-state EEG features, particularly phase reset rates; these findings provide new insights into the detection of early depression (Morita et al., 2023). The potential of PLV to detect abnormal neural synchrony patterns in patients with MDD has been demonstrated. Moreover, resting-state EEG has been investigated for the detection of MDD, and the coherence feature has been identified as a reliable and effective solution for EEG-based detection of MDD (Wu et al., 2021). Previous studies have also demonstrated differences in EEG functional connectivity between MDD patients and healthy individuals. Alpha-band functional connectivity in the default mode network (DMN) can predict depression severity and is more prominent in MDD patients than in healthy individuals (Simmatis et al., 2023). A previous study found that depressed patients had significantly reduced resting PLV-based functional connectivity in the delta band compared with healthy controls (Huang S. S. et al., 2023). Another study identified specific EEG features in MDD patients (e.g., lower alpha and higher gamma phase lag index-based connectivity) compared with healthy individuals (Huang Y. et al., 2023).

The resting state serves as a dynamic substrate for brain activity, encompassing a range of operational modes from sensory processing to attention. Previous research indicated that 60 to 80% of the brain’s energy budget was dedicated to supporting neuronal communication during the resting state, underscoring the predominance of this activity in brain function (Raichle, 2006). The DMN plays a crucial role in understanding resting-state activity and provides insights into the neural mechanisms of neurological disorders (Mohan et al., 2016). Low-resolution brain electromagnetic tomography (LORETA) is capable of using EEG data to determine the characteristics of resting-state networks across intrinsic frequency bands (Aoki et al., 2015). A previous study using LORETA demonstrated increased resting-state current source density in the frontal regions across the delta, theta, and beta bands (Korb et al., 2008). Another study identified decreased spectral power activity in the right middle temporal gyrus compared with healthy controls (Lubar et al., 2003).

Several biomarkers reflect the significant effects of depression on brain activity and can be used in its diagnosis. However, most studies have focused on differences in EEG characteristics between MDD patients and healthy controls, and it remains unclear whether there are differences in the EEG characteristics of healthy individuals with different depressive states. In our previous study, we reported differences in EEG characteristics among healthy individuals with varying depressive states who were exposed to visual stimuli (Li et al., 2023). This study highlighted that individuals in a more severe depressive state exhibited a decrease in P300 amplitude and distinct brain activity patterns in the frontal and parietal regions. The present study builds on this research by exploring significant differences in EEG characteristics among healthy individuals during the resting state. While our previous research explored EEG differences during specific tasks, further investigation is essential to understand how the brain functions differently in the resting state. This would potentially add new perspective on how depressive states affect neural activity. Additionally, resting-state EEG is less demanding for participants, which might make it more broadly applicable to the early detection of depression. Therefore, we investigated brain activity of healthy individuals in the resting state and hypothesized that this continuum might be present in the resting-state neural activity of healthy individuals.

In this study, we investigated whether there are significant differences in the neural activity of healthy individuals with different depressive states during the resting state. We assessed participants’ depressive states using the Beck Depression Inventory Second Edition (BDI-II), and EEG activity was recorded during the experiment. We investigated whether participants with more severe depressive states had reduced theta-band power, increased delta-band power, and lower power ratios relative to participants with less severe depressive states. We also investigated whether there was frontal-parietal impairment of functional neural connectivity, based on a PLV analysis of participants with different depressive states.

## 2 Materials and methods

### 2.1 Participants

We conducted further investigations using the same participants as in our previous study (Li et al., 2023). The participants comprised 46 individuals (female, *n* = 16; male, *n* = 30; mean age, 22 ± 3 years) with no history of depression diagnosis or ongoing medication. Ethical approval for this study was obtained through the Ethical Review of Human Subject Research of the Tokyo Institute of Technology (Approval Number: A20202) and Ethical Review of Human Research of Tokai University (Approval Number: 21134). The present study included 43 participants from the previous study population; 3 participants were excluded due to either incomplete datasets or outlier responses relative to other participants in the previous experimental task.

The severity of the depressive states of participants was evaluated using the Beck’s Depression Inventory (BDI-II), a 21-question inventory that probes into participants’ life and psychological conditions over the prior two-week period. Responses were scored on a 0–3 scale for each question, resulting in a total score of 0–63. Lower scores indicated less severe depressive symptoms. Given the non-clinical nature of the participants, the BDI-II scores in this study were generally low with limited variability (mean score: 6.413; standard deviation: 4.773). All participants completed the BDI-II questionnaire via Google Forms prior to the EEG experimental procedures.

### 2.2 Experimental design

The overall experiment consisted of three sections: depression state assessment, resting-state EEG, and EEG during emotional visual stimuli. In our previous study, we reported EEG characteristics during emotional visual stimuli, which were measured after resting-state EEG. While the present study used the depressive states results from the BDI-II questionnaire data, it shifted the focus to explore how depressive states are reflected in resting-state EEG.

The experiment design was as follows. First, depression was assessed using the BDI-II questionnaire, which was followed by placement of the EEG electrodes, explanation of the experimental procedure, and the EEG measurement session. For the EEG measurement session, a “+” mark was displayed in the center of the screen monitor to guide the participants’ gaze, during which time they were instructed to clear their minds as much as possible. To ensure the length of the data, we recorded EEG data for approximately 1.5 min. The experiments were conducted in a bright soundproof room, with the participant sitting comfortably on a chair and their head placed on a chin rest to prevent head movement.

### 2.3 Data measurement and analysis

Collection of EEG data was performed using the Polymate Pro MP6100 amplifier (Miyuki Giken Co., Tokyo, Japan), which recorded from 21 electrodes based on the International 10–20 system at a sampling frequency of 500 Hz, including the vertical electrooculogram (VEOG) and horizontal electrooculogram (HEOG). We ensured that the electrode impedance did not exceed the recommended value of 50 kΩ.

Data processing was conducted using a MATLAB-based script in conjunction with the EEGLAB toolbox. Raw signals were initially referenced to AFz during the collection phase and then re-referenced to the average of all EEG electrodes during pre-processing. A 0.5–50-Hz bandpass filter was implemented. The Infomax Independent Component Analysis algorithm was used to remove eye movement-, blink-, muscle-, and heart-related artifacts from EEG recordings.

#### 2.3.1 Analysis of PSD and power ratio

In this study, the PSD of EEG signals was computed using the pwelch function in MATLAB. Welch’s algorithm implements a periodogram-based spectral estimation method, using fast Fourier transform (FFT). This technique involves dividing the EEG time series into eight segments with 50% overlap. Each segment is then windowed with a Hamming window to minimize spectral leakage. The FFT is applied to each windowed segment to obtain periodograms, which are then averaged to determine the estimated PSD (Welch, 1967). The PSD was calculated for the preprocessed EEG recordings, utilizing the 1-min-long EEG data from each participant. To assess temporal fluctuations in resting-state EEG, the 1-min recording was segmented into six 10-s intervals, allowing for a nuanced PSD analysis across distinct frequencies and timeframes. Calculations were confined to specific frequency ranges: delta (1–4 Hz), theta (4–8 Hz), alpha (8–13 Hz), beta (13–30 Hz), and gamma (30–50 Hz)^2^. Average frequency-specific power values were computed to characterize the power spectrum within each band. Subsequently, the relative power for each frequency band was ascertained by normalizing the absolute power to the total power spectrum for each individual participant. To discern the influence of depressive states, statistical analyses were undertaken across three distinct groups, with adjustment for multiple comparisons.

For power ratios, we calculated the alpha–theta ratio (ATR), ABR, and TBR for each participant, and then conducted correlation analyses of these three power ratios separately to determine their correlation with the BDI-II score.

#### 2.3.2 Analysis of PLV

PLVs were calculated for each subject to quantify the synchronization strength between electrode pairs on the scalp in order to gain insight into the functional connectivity of the brain. PLVs range from 0 to 1, with values closer to 1 representing stronger synchronization. This analysis aimed to understand how the depressive state affects the functional connectivity between different areas.

For calculation of the PLV between two electrodes, the process begins with bandpass filtering of the data from each electrode within a specific frequency range. Subsequently, the Hilbert transform is applied to these filtered signals. This transformation converts the real-valued EEG signals into complex-valued analytic signals, which reveal the instantaneous phase of the EEG signal at each time point. In the next step, the instantaneous phase is extracted for each signal, and the phase difference between the two signals at each time point is calculated. Finally, the computation of the PLV is carried out by taking the mean of the exponential values of the complex phase differences over time, which quantifies the degree of phase synchronization between the two signals (Aydore et al., 2013).

The equation for the PLV between two electrodes is as follows:

$$P\,L\,V_{p\,q}^{f}=\left|\frac{1}{N}\,\sum_{t=1}^{N}e^{i\,\left(\phi_{p}^{f}\,\left(t\right)-\phi_{q}^{f}\,\left(t\right)\right)}\right|.$$

Here,

1. $\phi_{p}^{f}\,\left(t\right)$: instantaneous phases of signals from frequency band *f* and electrodes *p* at time point *t*.
2. $\phi_{q}^{f}\,\left(t\right)$: instantaneous phases of signals from frequency band *f* and electrodes *q* at time point *t*.
3. *N*: total number of time points in the signal.
4. The computation involves summing the exponential of the phase differences at all time points, followed by calculating the absolute value of this average to yield the PLV.

**FIGURE 1:**
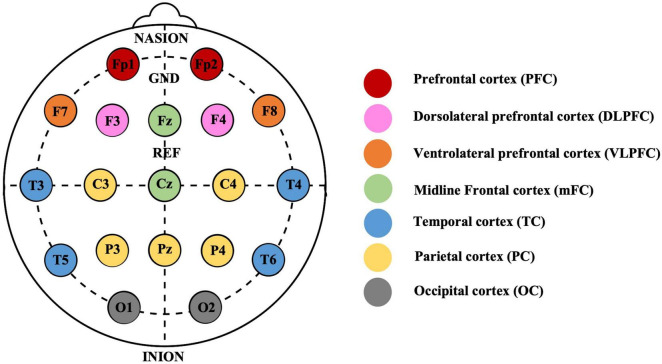
Electrodes placement using International 10–20 system and the electrodes corresponding to different areas. The 19 electrodes are categorized into 7 distinct brain regions, denoted by color-coded circles. Each region is integral to subsequent Phase Locking Value (PLV) analysis, which examines the neural connectivity across different areas of the brain.

We calculated PLVs for each frequency band and between each electrode pair for each subject and normalized them to ensure comparability across participants.^3^ We employed Min-Max normalization to scale the values, which transformed the data into a standardized range of 0 to 1. This normalization was achieved by subtracting the minimum value from each data point and then dividing the result by the difference between the maximum and minimum values. We then divided the brain into different regions and quantified the synchronization strength by calculating the average of the connection strength of one electrode to all electrodes in a specific region. We divided 19 electrodes based on the 10–20 system as follows (Figure 1): the prefrontal cortex (PFC) region, containing Fp1 and Fp2; the dorsolateral prefrontal cortex (DLPFC) region, containing F3 and F4; the ventrolateral prefrontal cortex (VLPFC) region, containing F7 and F8; the midline frontal cortex (mFC) region, containing Fz and Cz; the temporal cortex (TC) region, containing T3, T4, T5, and T6; the parietal cortex (PC) region, containing C3, C4, P3, P4, and Pz; and the occipital cortex (OC) region, containing O1 and O2 (Chai et al., 2019). We then performed a correlation coefficient analysis to explore the relationship between depressive states and the strength of neural synchronization across different electrodes and regions.

#### 2.3.3 Analysis of source localization

Resting-state EEG data recorded from 19 scalp electrodes were analyzed via standardized low-resolution electromagnetic tomography (sLORETA) for source localization (Pascual-Marqui, 2002). For each participant, 60 s of resting-state EEG data was utilized for cross-spectral computations. Functional independent component analysis was employed to determine the spectral density of electric neuronal generators across 6239 cortical voxels within the seven classical EEG frequency bands: delta (1–4 Hz), theta (4–8 Hz), alpha (8–12 Hz), beta (12–30 Hz), and gamma (30–50 Hz). This analysis aimed to identify specific regions and frequency bands associated with the resting-state network (Herrmann et al., 1979). Statistical evaluations were performed on the coefficients of 15 components to determine how various depressive states influence network utilization. A pairwise *t*-test was conducted among the three groups, with multiple testing corrections applied through statistical nonparametric mapping (SnPM) using 5000 permutations.

## 3 Results

### 3.1 Distribution of depressive states

**FIGURE 2:**
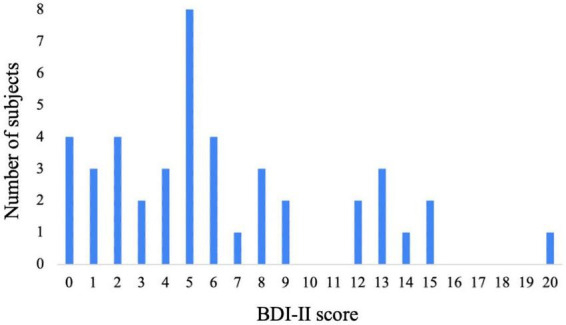
Distribution of BDI-II scores.

The distribution of participants’ depressive states is shown in Figure 2 (Li et al., 2023). Participants’ depressive states were assessed using the BDI-II. The maximum score of the BDI-II questionnaire is 63, but because the participants were healthy individuals who were not diagnosed with depression, the maximum score among the population in this study was 20. According to our previous study (Li et al., 2023), participants with a score of 0–3 were categorized into the low depressive state group (*n* = 13), those with a score of 4–9 were categorized into the medium depressive state group (*n* = 21), and those with a score of 10–20 were categorized into the high depressive state group (*n* = 9).

### 3.2 PSD and power ratio

**FIGURE 3:**
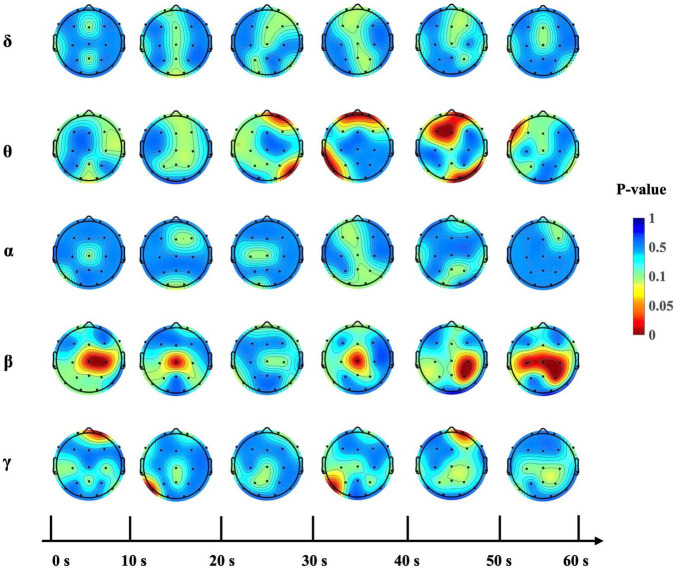
*P*-value topography across time intervals by frequency band. Topographic maps displaying the statistical significance of PSD differences among depressive states on 1-min EEG, segmented into six 10-s intervals. The color gradient, with red indicating lower *p*-values, shows the results of the one-way analysis of variance (*p* < 0.05).

PSD analysis was conducted to evaluate the impact of depressive states on EEG spectral power across different frequencies. The PSD serves as an indicator of the distribution of signal power among frequency components. Subsequently, a one-way analysis of variance was applied to determine the influence of depressive states on resting-state EEG activity. The findings are presented in Figure 3. Significant differences were observed mainly in the theta and beta bands across various time intervals. From 0 to 10 s, notable differences were observed in central beta power at Cz and C4. From 10 to 20 s, notable differences were observed in central beta power at Cz. From 20 to 30 s, notable differences were observed in frontal theta power at Fp2. From 30 to 40 s, notable differences were observed in frontal theta at Fp1 and Fp2, and central beta powers at Cz. From 40 to 50 s, notable differences were observed in frontal theta at Fp2, F3, and Fz, and parietal beta powers at C4 and P4. From 50 to 60 s, notable differences were observed in central and parietal beta powers at F7, Cz, C3, C4 and P4. Results of the statistical analysis are given in Table 1.

**TABLE 1:** Statistical analysis of PSD by time interval, brain region, and frequency band.

Time interval (s)	Brain region	Frequency band	Statistic	*F*-Value	*p*-Value	Effect size (η^2^)
0–10	Cz	Beta	F(2, 40)	4.0	0.038	0.17
	C4	Beta	F(2, 40)	3.5	0.038	0.15
10–20	Cz	Beta	F(2, 40)	3.5	0.039	0.15
20–30	Fp2	Theta	F(2, 40)	9.2	0.001	0.31
30–40	Fp1	Theta	F(2, 40)	5.5	0.031	0.22
	Fp2	Theta	F(2, 40)	4.1	0.031	0.17
	Cz	Beta	F(2, 40)	4.2	0.023	0.17
40–50	Fp2	Theta	F(2, 40)	3.6	0.037	0.15
	F3	Theta	F(2, 40)	5.3	0.023	0.21
	Fz	Theta	F(2, 40)	4.4	0.023	0.18
	C4	Beta	F(2, 40)	4.3	0.036	0.18
	P4	Beta	F(2, 40)	3.6	0.036	0.15
50–60	F7	Beta	F(2, 40)	3.9	0.029	0.16
	Cz	Beta	F(2, 40)	6.1	0.012	0.23
	C3	Beta	F(2, 40)	4.4	0.019	0.18
	C4	Beta	F(2, 40)	5.4	0.012	0.21
	P4	Beta	F(2, 40)	5.3	0.012	0.21

**FIGURE 4:**
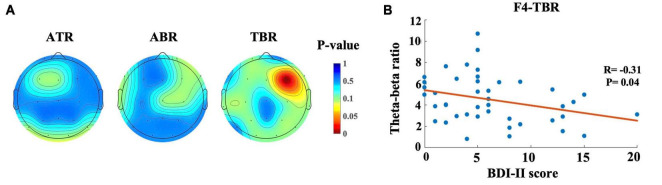
Topographic maps and scatter plot for correlation between BDI-II score and power ratio. **(A)** Topographic mapping of correlation for Alpha-theta ratio (ATR), Alpha-beta ratio (ABR) and Theta-beta ratio (TBR). The red color indicates a smaller *p*-value (*p* < 0.05), which represents the significant correlation between BDI-II score and the power ratio, **(B)** Relationship between the BDI score and TBR. Each dot represents the TBR of one participant.

Figure 4A shows the topographic mapping of correlation for ATR, ABR, and TBR. In this figure, red indicates a significant correlation between BDI-II score and power ratio. This correlation was found in the TBR in the left frontal area (*R* = −0.31, *p* < 0.05), as shown in Figure 4B.

### 3.3 Phase locking value

**FIGURE 5:**
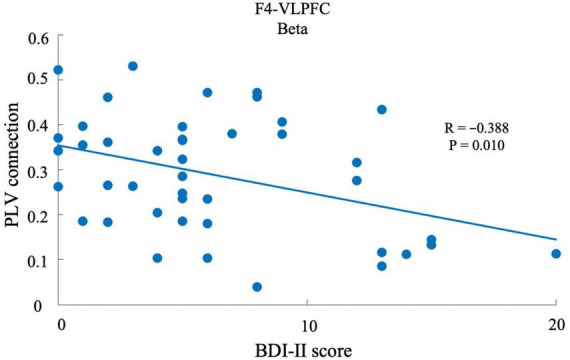
Relationship between the BDI-II score and PLV strength. The x-axis shows the BDI-II score, with a higher score associated with a higher depressive state. The y-axis shows the PLV strength between F4 and VLPFC, which was defined as the mean PLV strength between one electrode and the electrodes of an area. Each dot represents the average PLV strength of one participant.

We performed a correlation coefficient analysis to explore the relationship between depressive state and neural synchronization strength. For example, as shown in Figure 5, when the connection region was VLPFC and the band was the beta band (13–30 Hz), a relationship between BDI-II score and the connection strength of F4, with respect to beta band in VLPFC region, was demonstrated (*p* < 0.05). This indicates that the strength of neural synchronization in the beta band of the F4 with respect to the VLPFC region was significantly negatively correlated with the depressive state. This analysis was performed for each electrode and each specific frequency, and was transformed into topographic maps to show the distribution and strength of the correlation between depressive state and synchronization intensity across the scalp.

**FIGURE 6:**
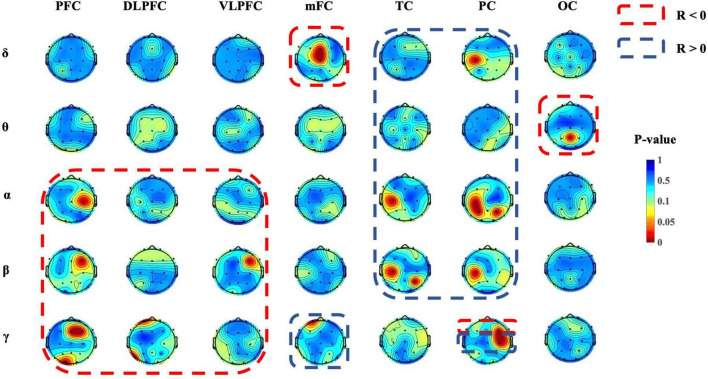
Topographic mapping of significant correlations in the PLV for different regions. Red color indicates lower *p*-values (*p* < 0.05), which represent a significant correlation between the BDI-II score and the PLV strength of a certain area and frequency.

As shown in Figure 6, significant correlations were observed between the strength of brain connections in various regions and depressive states across different frequency bands. For the delta band, there were notable correlations involving the connection strength within the mFC region and between the C3 and PC regions in relation to depressive states. For the theta band, connections between the Pz and OC regions showed a significant relationship with depressive states. For the alpha band, the connectivity strength between the C4 and PFC regions, between the C3 and TC regions, and within the PC region (specifically at locations C3, P3, and P4) were all significantly correlated with depressive states. For the beta band, connection strengths that showed significant correlations with depressive states included those between the F4 and PFC regions, the F4 and VLPFC regions, the C3 and TC regions, the C3 and PC regions, and the P4 and TC regions. For the gamma band, the connections between the PFC region and Fz, F4, and O1, as well as connections from the DLPFC region to Fp1 and T5, showed significant associations with depressive states. Additionally, connections from the mFC region to Fp1 and within the PC region to F4 and C4 also presented notable correlations with depressive states. Results of the statistical analysis are given in Table 2.

**TABLE 2:** Correlation between EEG connectivity and depressive state across different frequency bands.

Frequency band	Connection between regions	Correlation co-efficient (*R*)	*p*-value
Delta	mFC intra-region	−0.3	0.05
	C3 and PC	0.32	0.04
Theta	Pz and OC	0.36	0.02
Alpha	C4 and PFC	−0.35	0.02
	C3 and TC	0.33	0.03
	PC intra-region (C3)	0.32	0.04
	PC intra-region (P3)	0.33	0.03
	PC intra-region (P4)	0.3	0.05
Beta	F4 and PFC	−0.35	0.02
	F4 and VLPFC	−0.39	0.01
	C3 and TC	0.3	0.05
	C3 and PC	0.38	0.01
	P4 and TC	0.3	0.05
Gamma	Fz and PFC	−0.31	0.04
	F4 and PFC	−0.45	<0.01
	O1 and PFC	−0.31	0.04
	Fp1 and DLPFC	−0.36	0.02
	T5 and DLPFC	−0.35	0.02
	Fp1 and mFC	0.43	<0.01
	F4 and PC	−0.35	0.02
	C4 and PC	0.35	0.02

### 3.4 Source localization

**FIGURE 7:**
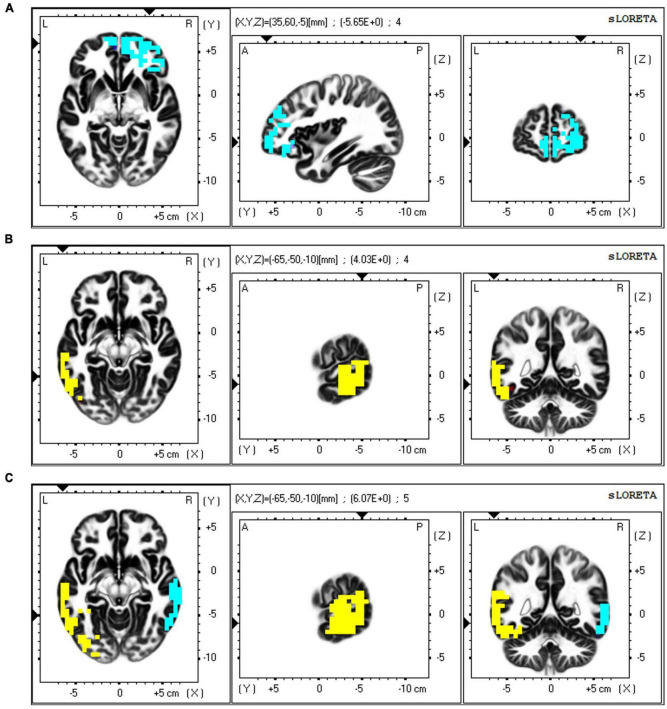
Resting state brain network analysis using standardized low resolution electromagnetic tomography method (sLORETA) source localization. **(A)** The beta band’s spatial distribution is linked to the functional independent component displaying the highest significant difference across the three depressive state groups, with the maximum theta band location (positive, color-coded yellow) identified in the frontal areas, **(B)** The beta band’s spatial distribution is linked to the functional independent component displaying the highest significant difference across the three depressive state groups, with the maximum beta band location (positive, color coded yellow) identified in the temporal areas, **(C)** The gamma band’s spatial distribution is linked to the functional independent component displaying the highest significant difference across the three depressive state groups, with the maximum gamma band location (positive, color-coded yellow; negative, color-coded blue) identified in the temporal areas. Horizontal (left), sagittal (middle), and coronal (right) sections through the voxel with the maximal *t*-statistic are displayed.

The neural substrates underlying the variations in resting neural networks among depressive states were examined using sLORETA, employing a one-tailed significance test. The exceedance proportion test from sLORETA determined significant component differences between conditions (*p* < 0.05). When comparing the low and high groups, the exceedance proportion test indicated a threshold of −2.100 corresponding to a *p*-value of 0.075; as shown in Figure 7A, significant beta-band variations were localized to the right middle frontal gyrus (*t* = 2.33, MNI coordinates: *X* = 35, *Y* = 60, *Z* = −5). When comparing the middle and high groups, beta-band differences were localized to the left middle temporal gyrus (*t* = 2.88, MNI coordinates: *X* = −65, *Y* = −50, *Z* = −10), with a threshold of −2.018 linked to a *p*-value of 0.015, as shown in Figure 7B. Differences in the gamma band were localized to the left middle temporal gyrus (*t* = 2.12, MNI coordinates: *X* = −65, *Y* = −50, *Z* = −10) and the right middle temporal gyrus (*t* = 2.12, MNI coordinates: *X* = 65, *Y* = −30, *Z* = −10; Figure 7C).

## 4 Discussion

This study investigated the effects of different depressive states on neural activity in healthy individuals by analyzing the changes in power spectrum density, power ratio, and region-based phase locking value with respect to the depressive state during the resting state. In terms of PSD, the theta band of the prefrontal cortex showed a tendency to decrease as the depressive state increased, and the delta band showed an upward trend as the depressive state increased. In terms of power ratio, the TBR of the right frontal region showed a decreasing trend with an increasing depressive state. In terms of functional connections, significant correlations were observed within the frontal and parietal region. In terms of source localization by sLORETA, frontal beta, temporal beta, and temporal gamma activities were identified.

The PSD results indicated an increasing trend in the delta band in the prefrontal area in the resting state, but this difference was not statistically significant. This may suggest a more complex or different neural mechanism than hypothesized. In previous studies, resting-state EEG delta power was reported to be associated with psychological pain in adults with a history of depression (Meerwijk et al., 2015). Resting-state EEG delta power has been found to have predictive utility in predicting the response of individuals with depression to cognitive behavioral therapy (Schwartzmann et al., 2023). In addition, previous studies have shown that for patients with depression, higher delta bands in the left frontal region during resting states are associated with greater self-disgust (Amico et al., 2023) and with greater cognitive load associated with visual input during eye-open resting (Kan et al., 2017). Higher delta power in the left temporal region is associated with different thought wandering processes (Spironelli et al., 2021). Therefore, delta power that shows an increasing tendency with increasing depressive states might be associated with unstable mental states.

The theta band of the resting state tends to increase with the severity of the depressive state in the frontal area, and a significant difference was found in the prefrontal cortex. Resting-state theta activity has been linked to information content-specific coding levels during response inhibition (Pscherer et al., 2022). A higher resting theta activity was found to be associated with a stronger N2 peak during successful response inhibition for no-go tasks, suggesting that the resting-state theta activity forms the foundation for a neural state that is helpful in enhancing inhibitory control mechanisms more effectively (Pscherer et al., 2022). In our previous study, the low depressive state group showed a stronger N2 peak during stimuli processing (Li et al., 2023), and the present study found stronger resting theta power in the low depressive state group. Therefore, the higher resting theta power of the low-depression state may indicate better control over external stimuli, and thus maintenance of a stable mental state. Another study demonstrated a negative correlation between theta power of the right central region and depressive states in patients with depression (Gao et al., 2023). Therefore, the PSD results of our present study may indicate that neural activity is modulated by depressive states and that it is derived from a state of psychological distress and a lack of inhibition of negative information.

The TBR at the F4 electrode was found to be negatively related to depressive states. A decreased TBR has been proposed as a potential biological marker of depression, with significant differences observed across various electrode ranges, indicating alterations in EEG power ratios in the resting state of depression (Chang and Choi, 2023). In addition to the results of scalp-recorded EEG activity, the results of the source localization analysis provided further support for a more localized difference in the beta band in the frontal lobe among the three depressive state groups, suggesting that different levels of depressive states affect the resting-state network in the frontal region, specifically the middle frontal gyrus.

The PLV results demonstrate the correlation between the strength of PLV connections and the depressive state. In the present study, a negative correlation within the frontal lobe was found in the delta, beta, and gamma bands. In patients with MDD, alterations in delta-band connectivity have been observed, with reduced resting brain connectivity in the delta band relative to healthy controls (Qiu et al., 2022). Compared with healthy controls, decreased beta-band functional connectivity has been found in MDD patients during emotional stimuli processing (Zuchowicz et al., 2019). A negative correlation between the frontal and parietal lobes could be found in the alpha and gamma bands, and a negative correlation between the frontal and temporal lobes could be found in the gamma band. Results of the source localization analysis also confirmed a specific variation of the gamma band in the temporal lobe across three depressive state groups, suggesting that varying degrees of depressive states influence the resting-state network in the temporal region, particularly in the middle temporal gyrus. A previous study found that the average functional connectivity of the alpha band during the resting state showed a negative correlation with the severity of depression (Shim et al., 2018). Research has also shown that resting-state EEG activity, particularly in the beta band, is a marker of the strength of frontoparietal connections, which are associated with attentional performance (Rogala et al., 2020). Additionally, increased theta and alpha connectivity for resting EEG have been identified in dysphoria, which may represent the risk of depression (Dell’Acqua et al., 2021). Negative correlations of resting-state EEG functional connectivity in the frontal regions may indicate that synchronization or communication in frontal regions decreases with an increasing depressive state, and that the severity of the sub-threshold depressive state may be identified by the degree of reduction in functional connectivity in the frontal area.

Positive correlations within the parietal lobe were found in the delta, alpha, beta, and gamma bands, and between the parietal lobe and temporal lobe in the alpha and beta bands. Alterations in alpha-band connectivity have been observed in patients with MDD, with increased coherence primarily involving the prefrontal region (Leuchter et al., 2012). A previous study found that beta-band EEG functional connectivity in MDD patients was stronger than that in healthy controls in an eyes-closed resting state (Huang S. S. et al., 2023). Increased beta-band phase synchronization was found in the brain activity of MDD patients who did not respond to repetitive transcranial magnetic stimulation treatment (Zuchowicz et al., 2019). Increased resting gamma-band phase synchronization has been associated with the severity of depression, indicating a potential link between gamma-band connectivity and the characteristics of depressive symptoms (Yang et al., 2023). Additionally, deficits in gamma-band oscillations have been observed in other psychiatric conditions, including bipolar disorder, suggesting that gamma-band synchronization may be a relevant marker for understanding various mental health disorders (Tsai et al., 2023). The positive correlation in resting-state EEG functional connections in the parietal area indicates that the synchronization in the parietal region is strengthened with an increasing depressive state. Additionally, enhanced resting-state EEG connectivity in the posterior region was observed in patients with moderate to severe late-life depression relative to patients with mild depression, and was associated with attention deficits (Zeng et al., 2024). Leuchter et al. (2012) reported that, in comparison to healthy controls, unmedicated individuals with MDD had significantly higher overall coherence in the delta, theta, alpha, and beta frequency bands, indicating a loss of selectivity in resting functional connectivity. These results suggest that brain functional abnormalities in subthreshold depression are associated with changes in the functional connectivity in the frontal and parietal lobes, which may be important for the early detection and diagnosis of depression in the future.

This study has some limitations. While prior research has established the validity of classifying participants using BDI-II scores (Cox et al., 2001), the adequacy of the sample sizes of each of our groups remains uncertain. Future studies should aim to expand the number of participants in order to more robustly discern significant correlations with other biomarkers. Secondly, given that depression is more commonly observed in women (Piccinelli and Wilkinson, 2000), the fact that male participants accounted for approximately two-thirds of the study population represents a limitation. The under-representation of women may make the findings less applicable to the female population. To enhance generalizability, future studies should aim to address this by ensuring a more balanced sex distribution. The requirement of EEG for source localization is another limitation of this study. The analysis was conducted with 19-channel EEG recordings, which could provide a broad overview of brain activity. In a previous study, sources of resting-state EEG activity were estimated using 19-channel EEG recordings with LORETA (Aoki et al., 2015). However, a recent study suggests that a minimum of 64 electrodes is recommended for a robust source localization analysis (Hatlestad-Hall et al., 2023). Consequently, the source localization results obtained in this study should be considered a preliminary estimate. A more detailed source localization analysis using high-density EEG data is currently planned to enhance the accuracy and reliability of our findings.

In this study, we observed that the PSD in the theta band, the TBR, source localization tomography (sLORETA), and the PLV in the frontal and parietal lobes varied according to the depressive state in healthy individuals during a resting state. These findings suggest that depressive states may influence neural activity during resting states, potentially affecting cognitive functions. The PSD, power ratio, and connectivity within the neural circuits, particularly between the frontal and parietal lobes, could serve as an effective indicator and contribute to improving the understanding of neural activity during the resting state in healthy individuals. The identification of these neural patterns associated with depressive states could act as parameters for evaluating depressive states in healthy individuals, potentially serving as early markers for MDD.

## Data availability statement

The raw data supporting the conclusions of this article will be made available by the authors, without undue reservation.

## Ethics statement

The studies involving humans were approved by the Ethical Review of Human Subject Research of the Tokyo Institute of Technology and Ethical Review of Human Research of Tokai University. The studies were conducted in accordance with the local legislation and institutional requirements. The participants provided their written informed consent to participate in this study.

## Author contributions

PL: Formal analysis, Funding acquisition, Investigation, Methodology, Visualization, Writing−original draft, Writing–review and editing. MY: Data curation, Methodology, Writing–review and editing. DO: Data curation, Methodology, Writing–review and editing. HN: Conceptualization, Funding acquisition, Investigation, Methodology, Project administration, Supervision, Writing–review and editing. TY: Conceptualization, Funding acquisition, Investigation, Methodology, Project administration, Supervision, Writing–review and editing.

## Footnotes

1
http://www.jstor.org/stable/resrep28026.1

2
https://www.mathworks.com/help/signal/ref/pwelch.html

3
https://www.mathworks.com/help/signal/ref/hilbert.html
